# The somatic genetic architecture of human cancer: heterogeneity and the challenges for translational medicine

**DOI:** 10.1186/1479-5876-10-S2-A7

**Published:** 2012-10-17

**Authors:** Andy Futreal

**Affiliations:** 1 Genomic Medicine, MD Anderson Cancer Center, Houston, USA

## 

Advances in sequencing technology have led to an unprecedented opportunity to characterize the genomes of human cancers. Our work has been focused on characterizing intra- and intertumoral heterogeneity in several tumor types. Work on breast cancer has revealed substantial complexity of operative cancer genes, with marked diversity between cancers revealed by exome sequencing. Further work in both renal and breast cancer has begun to define the architecture of intratumoral heterogeneity – revealing evidence for substantial branched and, in some cases, convergent evolution within the same tumors. These data and others, particularly as they relate to the challenges of translation and genomics-base medicine will be discussed.

